# Disseminating findings from the Data Analysis with Privacy Protection for Epidemiological Research (DAPPER) workshop

**DOI:** 10.23889/ijpds.v1i1.301

**Published:** 2017-04-19

**Authors:** Rebecca Wilson, Paul Burton, Innes Asher, Kim Carter, Adriana Castelli, David Cumin, Chris Evelo, Lesley Stewart, Kelvin Tsoi, Emma White

**Affiliations:** University of Bristol; University of Auckland; University of Western Australia; University of York; Maastricht University; Chinese University of Hong Kong; University of Southampton

## Objectives

The effective exploitation of what are often called big data is increasingly important. They provide the evidence in evidence-based health care and underpin scientific progress in many domains including social/economic policy. Typically, an optimal analysis involves working directly with microdata; i.e. the detailed data relating to each individual in the dataset. But there are many ethico-legal and other governance restrictions on physically sharing microdata. Furthermore, researchers or institutions may have an extensive intellectual property investment in complex microdata and although keen for other researchers to analyse their data they may not wish to give them a physical copy. These restrictions can discourage the use of optimum approaches to analysing pivotal data and slow scientific progress. Data science groups across the world are exploring privacy-protected approaches to analysing microdata without having to physically share the data.

## Approach

A two day international workshop was arranged focussing on privacy protected approaches to data analysis - particularly federated analysis where raw data remain at their original site of collection. The workshop considered the range of approaches that exist, and those that are currently being developed. It explored the strengths, weaknesses, opportunities and challenges associated with these methods and identified situations where specific approaches have a particularly important role. The workshop included a number of practical sessions where potential users could watch demonstrations of the various approaches in action and run analyses themselves.

## Results

The Data Analysis with Privacy Protection for Epidemiological Research (DAPPER) workshop was held 22-23rd August 2016, Bristol. We report back to the broader community on the outcomes of this workshop that focussed on exploring current approaches, tools and technical solutions that facilitate sensitive data to be shared and analysed.

## Conclusion

The workshop has helped map out key opportunities and challenges and assisted potential users, developers and other stakeholders (e.g. funders/journals) to recognise the strengths and weaknesses of different privacy protected analytic approaches. The workshop will encourage further methodological work in this field and better informed application of existing methods.

